# Minimally Invasive Robotic Laser Corpus Callosotomy: A Proof of Concept

**DOI:** 10.7759/cureus.1021

**Published:** 2017-02-10

**Authors:** Harminder Singh, Walid I Essayed, Sayantan Deb, Caitlin Hoffman, Theodore H Schwartz

**Affiliations:** 1 Neurosurgery, Stanford University Medical Center; 2 Department of Neurosurgery, Weill Cornell Medical College, New York Presbyterian Hospital, New York; 3 Medical School, Stanford University School of Medicine; 4 Weill Cornell Brain and Spine Center, Weill Cornell Medical College, New York Presbyterian Hospital, New York

**Keywords:** corpus callosotomy, minimally invasive, laser interstitial thermotherapy

## Abstract

**Introduction:**

We describe the feasibility of using minimally invasive robotic laser interstitial thermotherapy (LITT) for achieving an anterior two-thirds as well as a complete corpus callosotomy.

**Methods:**

Ten probe trajectories were plotted on normal magentic resonance imaging (MRI) scans using the Brainlab Stereotactic Planning Software (Brainlab, Munich, Germany). The NeuroBlate® System (Monteris Medical, MN, USA) was used to conform the thermal burn to the corpus callosum along the trajectory of the probe. The distance of the ideal entry site from either the coronal suture and the torcula or nasion and the midline was calculated. The distance of the probe tip from the dorsal and ventral limits of the callosotomy in the sagittal plane were also calculated.

**Results:**

Anterior two-thirds callosotomy was possible in all patients using a posterior parieto-occipital paramedian trajectory through the non-dominant lobe. The average entry point was 3.64 cm from the midline, 10.6 cm behind the coronal suture, and 9.2 cm above the torcula. The probe tip was an average of 1.4 cm from the anterior commissure. For a total callosotomy, an additional contralaterally placed frontal probe was used to target the posterior one-third of the corpus callosum. The average entry site was 3.3 cm from the midline and 9.1 cm above the nasion. The average distance of the probe tip from the base of the splenium was 0.94 cm.

**Conclusion:**

The directional thermoablation capability of the NeuroBlate® system allows for targeted lesioning of the corpus callosum, to achieve a two-thirds or complete corpus callosotomy. A laser distance of < 2 cm is sufficient to reach the entire corpus callosum through one trajectory for an anterior two-thirds callosotomy and two trajectories for a complete callosotomy.

## Introduction

Corpus callosotomy is an effective palliative surgical disconnection surgery for medically intractable non-focal epilepsy. Interrupting the interhemispheric propagation of the seizure reduces the magnitude as well as the frequency of the seizures, particularly the drop attacks [1-3]. The classic callosotomy is performed through a craniotomy located over the coronal suture and involves an interhemispheric approach, often resulting in entry into the lateral ventricle. Complications of the classic approach such as brain contusion, venous infarct, hydrocephalus, and infection can severely impede the prognosis of already disabled patients. In recent years, in order to decrease the invasiveness of such procedures, sporadic reports on minimally-invasive techniques using endoscopic-assisted procedures and gamma knife radiosurgery have been described or attempted [4-8]. In this paper, we describe the feasibility of using minimally invasive robotic laser thermotherapy for achieving an anterior two-thirds as well as a complete corpus callosotomy, thereby obviating the need for a craniotomy and approach-related complications.

## Materials and methods

The NeuroBlate® System (Monteris Medical, MN, USA)  was developed for thermoablation and coagulation of deep-seated lesions through interstitial delivery of laser power to the brain parenchyma. The delivery platform is a CO2-cooled fiber optic catheter, which is either 2.3 or 3.3 mm in diameter and can be placed into a predetermined target through either frameless or frame-based stereotactic systems. In the Monteris system, the laser can be directionally pulsed through a side-firing port to deliver controlled thermal energy, while the MRI scanner measures heat deposited in the target tissue. A robotic system rotates and withdraws or advances the laser probe to ablate a predetermined volume of tissue along the length of the probe trajectory.

**Figure 1:**
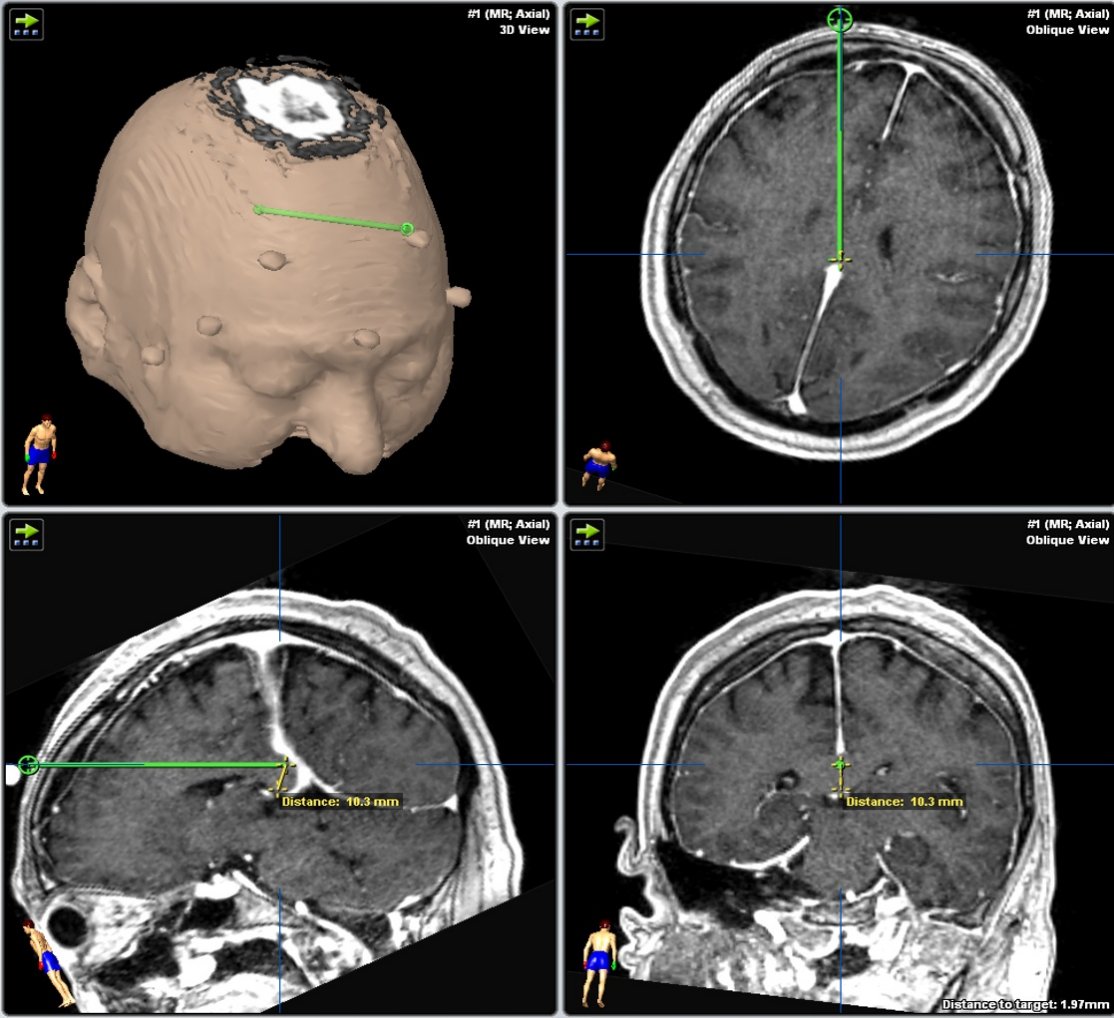
Trajectory planning of the anterior probe on Brainlab software. The distance from the tip of the probe to the inferior boundary of the splenium is measured.

**Figure 2:**
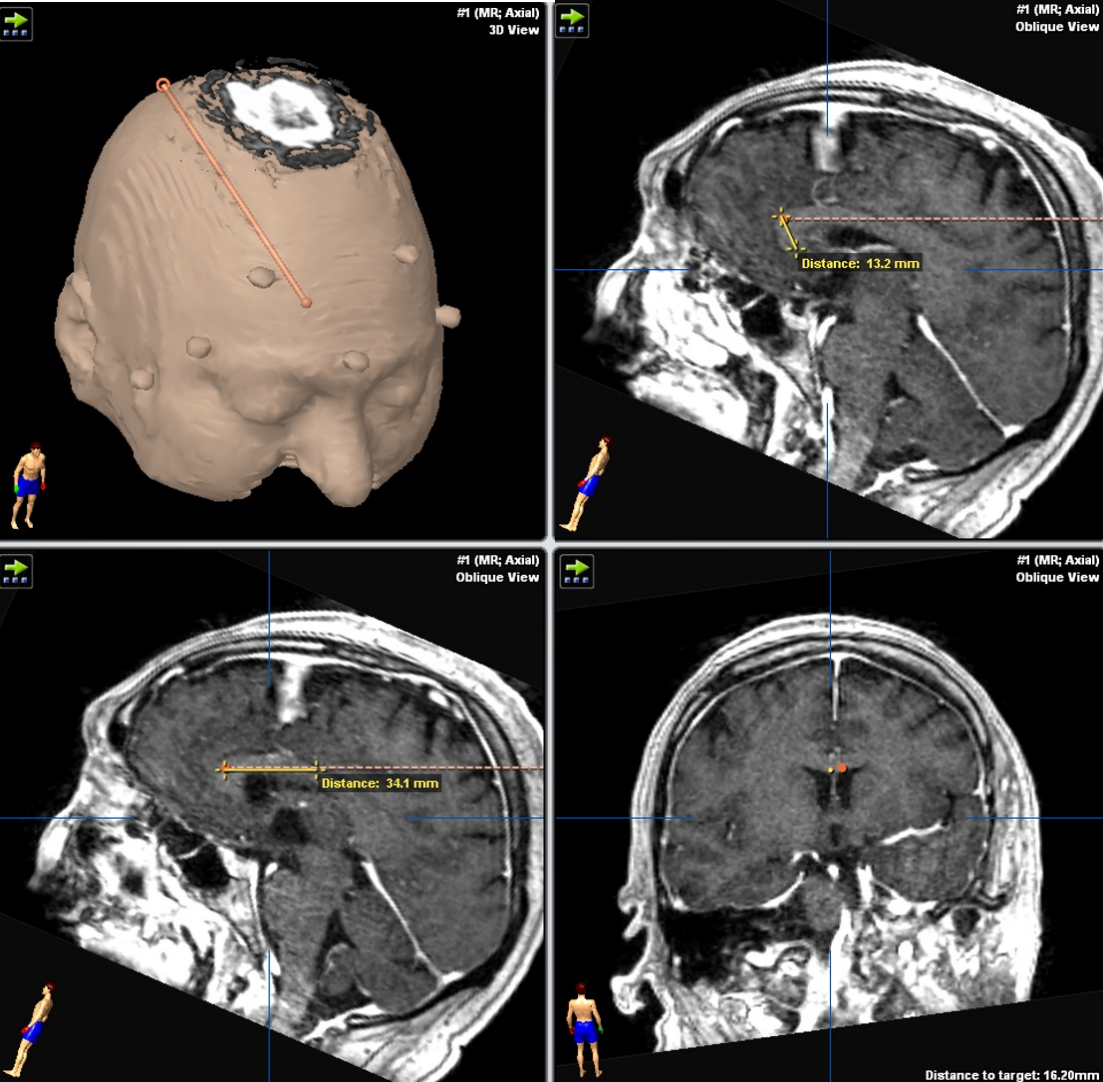
Trajectory planning of the posterior probe on Brainlab software. The distance of the tip of the probe to the inferior boundary of the genu is measured. Also, the distance from the genu to the entry point of the probe into the corpus callosum is measured.

**Figure 3:**
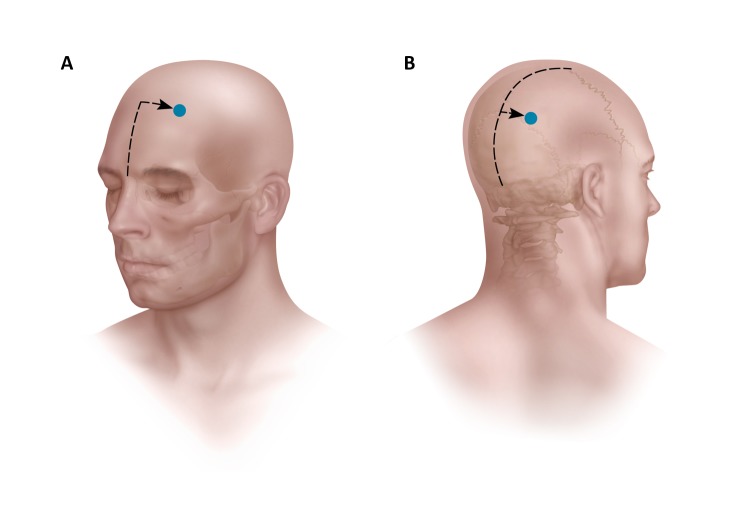
Artist rendition showing the entry point measurements of the anterior and posterior probes on the cranium. The anterior measurements are made from the nasion and midline. The posterior measurements are made from the torcula, the coronal suture, and midline.

Ten trajectories were plotted on normal MRI scans using the Brainlab Stereotactic Planning Software (Brainlab, Munich, Germany) (Figures 1-2). First, the volume of the callosotomy to be attained was defined on each MRI. In order to determine the optimal trajectory for achieving anterior two-thirds or complete callosotomy, we established a few guiding principles. First, the corpus callossum does not need to be ablated in the midline but can be ablated anywhere along the fiber tract before the fibers diverge, roughly 2 cm from midline on either side along the roof of the lateral ventricle. Second, the trajectory must not cross the plane of the pericallosal arteries to reduce the risk of an infarct. Third, the trajectory should not pass through the lateral ventricle to avoid creating a tract for egress of cerebrospinal fluid (CSF), although this latter principle is only a relative contraindication since passing stereotactic probes through the ventricles is considered safe. Fourth, the entry site should not be on the forehead for cosmetic purposes. Ideal trajectories for an anterior two-thirds callosotomy and a posterior one-third callosotomy were determined, and their entry sites were established relative to known landmarks, namely the sagittal and coronal sutures, the nasion, and the torcula. A single trajectory was always sufficient for an anterior two-thirds callosotomy, and two trajectories were sufficient for a complete callosotomy. Each patient case was considered for either an anterior two-thirds callosotomy using a posterior parietal approach or a complete callosotomy, combining the prior approach with a second frontal approach to reach the posterior one-third of the corpus callosum. Entry sites on opposite sides of the head were chosen to avoid collision of the two probes, so that both could be placed simultaneously to avoid having to return to the operating room to place a second probe, if intraoperative MRI is not available. Since the corpus callosum is a midline structure, either side can be chosen as an entry site. The measurements from the entry points, relative to classic superficial landmarks (nasion, coronal suture, torcula, and midline) were reported (Figure 3). The entry point of the posterior probe into the corpus callosum was measured from the genu (Figure 2). The distances between the posterior trajectory tip and the genu and the anterior trajectory tip and the splenium were also reported (Figures 1-2).

## Results

**Figure 4:**
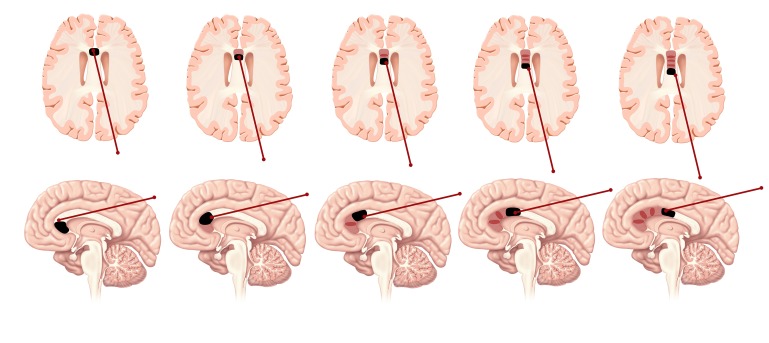
Placement of the posterior probe along the corpus callosum for an anterior two-thirds callosotomy. The probe is slowly retracted back as multiple thermal burns are executed (with MRI guidance) to cover the desired length of the corpus callosum.

Anterior two-thirds callosotomy was possible in all patients using a posterior parieto-occipital paramedian trajectory (Figures 2, 4). The average entry point on the cranium was 3.64 cm (2.9–4.2 cm) from the midline and either 10.6 cm (8.3–12.1 cm) behind the coronal suture or 9.2 cm (7.3–11.2 cm) above the torcula (Table 1, Figure 3). The average entry point of the probe into the corpus callosum was 4.09 cm (3.4–4.7 cm) behind the genu (Table 3). Thermotherapy at the genu is achieved by side-directing the laser inferiorly from the superoanterior part of the genu towards the anterior commissure. The posterior probe tip was an average of 1.4 cm (10.4–1.72 cm) from the anterior commissure (Table 3, Figure 2). 

**Figure 5:**
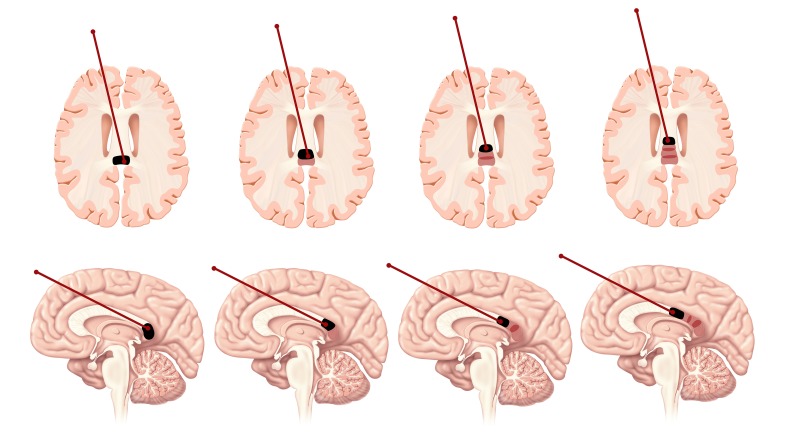
Placement of the anterior probe along the corpus callosum for a posterior one-third callosotomy.

When a complete callosotomy is desired, the complementary anterior trajectory was effective in targeting the posterior one-third of the corpus callosum (Figures 1, 5). The average entry point on the cranium was 3.3 cm (2.5–4.2 cm) from the midline and 9.1 cm (7.6–10.3 cm) above the nasion (Table 2). The entry point for the frontal trajectory should be set as high as possible so that the incision is behind the hairline, but also to facilitate the side burning of the posteroinferior part of the splenium (Figure 1). The average distance between the most posterior point reached by the probe and the inferior edge of the splenium was 0.94 cm (0.62–1.32 cm) (Table 3, Figure 1). The path should also be tailored to avoid the head of the caudate nucleus. The entry point into the corpus callosum should correspond to the previous parieto-occipital trajectory entry point so as to optimize corpus callosum coverage (Figure 1).

**Table 1:** Posterior trajectory entry point measurements (in cm).

	Above the Torcula	Behind the Coronal Suture	From the Midline
1	7.3	10.8	3.5
2	9.4	11	2.9
3	11.2	10.4	3.5
4	10.1	11.7	3.2
5	9.1	11.4	3.8
6	8.7	12.1	3.3
7	9.6	11.2	4.1
8	8.2	9.6	3.8
9	9.1	9.7	4.2
10	9.2	8.3	4.1
Mean	9.19	10.62	3.64

**Table 2:** Anterior trajectory entry point measurements (in cm).

	From the Nasion	From the Midline
1	8.2	2.9
2	10.3	2.5
3	9.8	3.1
4	8.2	3.7
5	7.6	3.9
6	9.6	4.2
7	8.8	3.5
8	9.3	4.1
9	9.3	3.2
10	9.9	2.5
Mean	9.1	3.36

**Table 3:** Corpus callosum (CC) entry point and distal measurements from the tip of the anterior and posterior probes (in cm). See Figure 1 and Figure 2 for illustration.

	Posterior Trajectory		Anterior Trajectory
	Distance from genu to entry of probe into CC	Distance from probe tip to genu	Distance from probe tip to splenium
1	4.25	1.49	0.62
2	4.26	1.41	0.97
3	4.21	1.39	1.05
4	3.81	1.27	0.72
5	3.70	1.61	1.19
6	4.70	1.04	0.62
7	3.41	1.32	1.03
8	4.18	1.45	0.82
9	4.62	1.63	1.11
10	3.85	1.72	1.32
Mean	4.09	1.43	0.94

## Discussion

Corpus callosotomy is a valuable surgical treatment option for patients with medically intractable epilepsy, especially effective in patients with drop attacks and seizures that rapidly generalize. However, this procedure is still associated with multiple morbidities. Some of these are approach-related while others are intrinsically associated with callosal fiber disconnection. Mutism and motor weakness can develop as well [2, 9-10].

Vagal nerve stimulation (VNS) is an alternative to corpus colostomy that can be used in the same group of patients with similar, albeit slightly worse, results but increased safety profile. The VNS works by decreasing the general susceptibility of the cortex to epilepsy [5, 9-10]. However, current data confirms the overall superiority of surgical corpus callosotomy to VNS for seizure control [1, 3, 5, 9-10].

The decision for a corpus callosotomy is made frequently in functionally impaired patients; thus, any surgery-associated complication can further undermine functional recovery. For the approach, the surgery usually necessitates a considerable median and paramedian craniotomy [2, 10]. Unroofing the superior sagittal sinus and dural opening can be associated with venous complications [9, 11]. Following the interhemispheric route, damage to the callosomarginal and pericallosal arteries can lead to ischemic incidents. Retraction or inadvertent injury to the cingulate gyrus and paracentral lobule can be associated with cognitive and motor complications respectively [2, 9-11].

To avoid these approach-related complications, recent minimally-invasive techniques have been reported. Tubbs, et al. first described an endoscopic callosotomy through an eyebrow incision in a cadaveric study [8]. In a subsequent cadaveric report, a frontal precoronal craniotomy was used to achieve a total endoscopic callosotomy [4]. In a recent report, Sood, et al. used the same precoronal route to successfully perform an endoscopic total callosotomy in four patients [7]. However, even if the above described technique represents a minimally invasive approach of performing a corpus callosotomy, it still necessitates a small craniotomy and a microscopic interhemispheric approach before switching to the endoscopic portion of the procedure.

Some reports describe the uses of radiosurgery to perform a partial or total callosotomy [5-6, 12]. Although the preliminary results of this minimally invasive technique are encouraging, a long latency of the effects limits the practicality in patients with severely debilitating seizures [2, 5-6, 12]. Further assessment and patient follow-up is also necessary to confirm long term results. Moreover, the long-term side-effects of ionizing radiation with such a large number of lesions required for callosotomy are unclear.

As an alternative to the above mentioned techniques, we assessed the feasibility of using minimally invasive robotic laser thermotherapy for performing a corpus callosotomy. In comparison with other available probes, the NeuroBlate® System allows a directional firing of the laser, which is appealing due to the curved shape of the corpus callosum. We found that the best-suited trajectories should be paramedian to avoid the superior sagittal sinus and its tributary veins, as well as to avoid the precuneal and cuneal sulci that project off the occipital falx. To reach the anterior two-thirds of the corpus callosum, the posterior trajectory should be directed high enough through the parieto-occipital lobe. Even if the probe trajectory transgresses functional occipital parenchyma, the small size of the probe decreases the risk for any significant visual impairment.

A parasagittal trajectory might endanger motor fibers and parenchyma, which is a risk that is also shared by microscopic callosotomy [1-2, 10-11], particularly when the disconnection is not strictly carried in the midline. However, the side-firing directional laser offered by the NeuroBlate® probe allows us to tailor the delivered heat as medial as possible across every point along the trajectory. The ability to direct the delivered laser thermotherapy can help reach curved segments of the corpus callosum such as the genu and splenium (Figures 1-2, 4-5).

The projected effects of the laser-delivered thermotherapy have only been evaluated in standard parenchyma where blood flow through surrounding vessels help in dissipating the heat. Similarly, the expected effect on the corpus callosum white matter can also be properly computed. The CSF flow in the closely related lateral ventricles also helps to dissipate the heat. The live feedback of the heat deposition from real time MRI-thermometry will help in adapting the existing model for treatment delivery.

## Conclusions

The directional thermoablation capability of the NeuroBlate® System allows for targeted lesioning of the corpus callosum, to achieve a two-thirds or complete corpus callosotomy. A laser distance of < 2 cm is sufficient to reach the entire corpus callosum through one trajectory for an anterior two-thirds callosotomy and two trajectories for a complete callosotomy. We present a proof of concept of the technique. Further clinical reports will be necessary to validate this technique as a realistic minimally-invasive alternative for corpus callosotomy.
